# Supplementary material for: Phosphorylation-induced conformation of β2-adrenoceptor related to arrestin recruitment revealed by NMR
Source: Nat Commun. 2018 Jan 15;9:194. doi: 10.1038/s41467-017-02632-8 (PMC5768704; doi:10.1038/s41467-017-02632-8)
Supplement: Supplementary file 1 — Supplementary Information [file 41467_2017_2632_MOESM1_ESM.pdf]

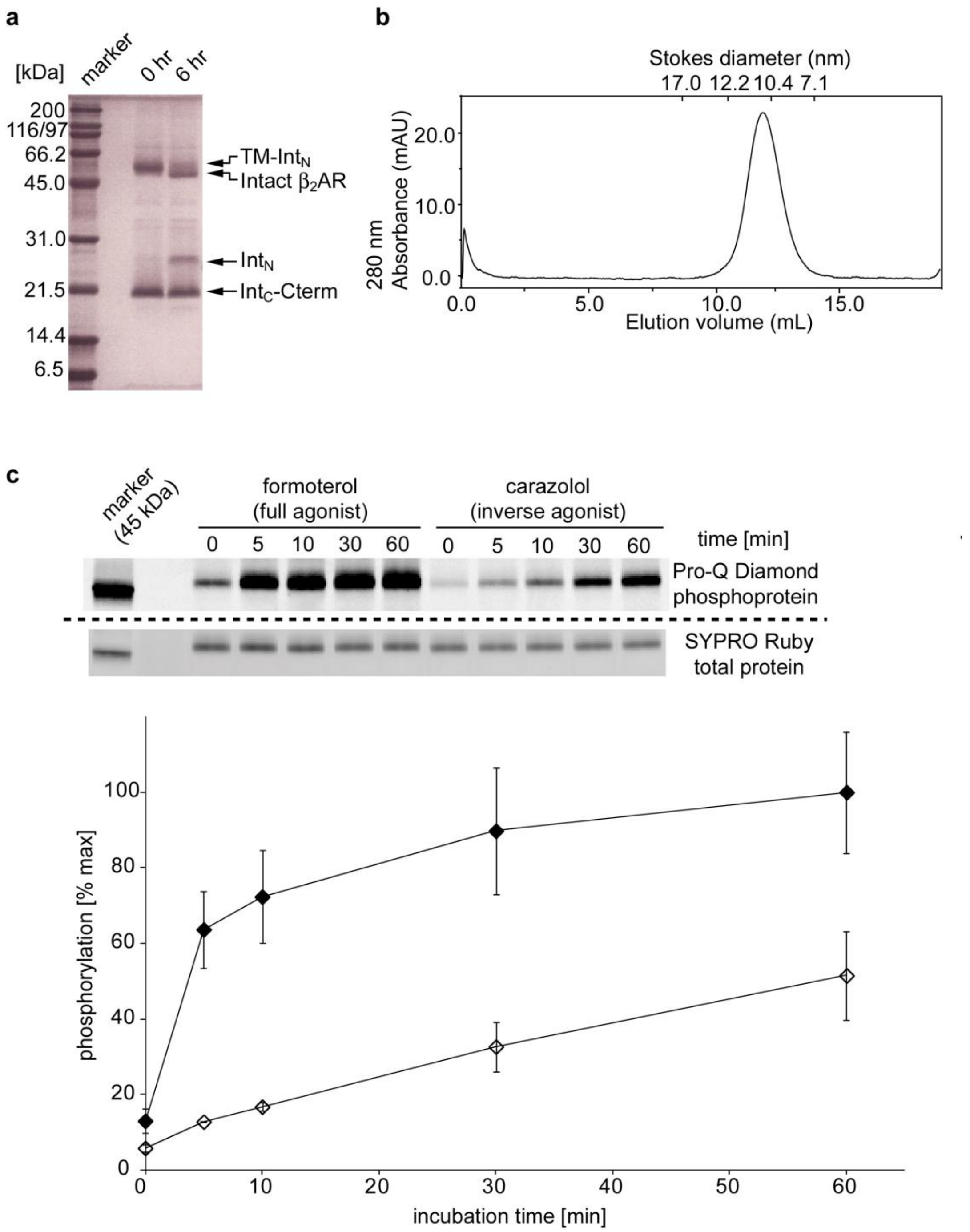

Supplementary Figure 1 Preparation and characterization of segmentally-labeled β2AR in rHDLs. (a) SDS-PAGE analysis of the reaction mixture of the protein *trans*-splicing reaction, before and after incubation. (b) Size exclusion chromatography analysis of the prepared β$_2$AR embedded in rHDLs. Elution volumes corresponding to 17.0, 12.2, 10.4, 7.1 nm Stokes diameters were determined by thyroglobulin, ferritin, catalase, and bovine serum

albumin standard, respectively. (c) GRK2-mediated phosphorylation of prepared β_2AR in rHDLs, in the full agonist-bound state and the inverse agonist-bound state. Reaction mixtures at the indicated time points were analyzed by SDS-PAGE with Pro-Q® Diamond and SYPRO®-Ruby staining. Error bars represent standard deviations of three independent experiments. Gel images are shown in the top panel. Uncropped gel images are shown in Supplementary Fig. 9.

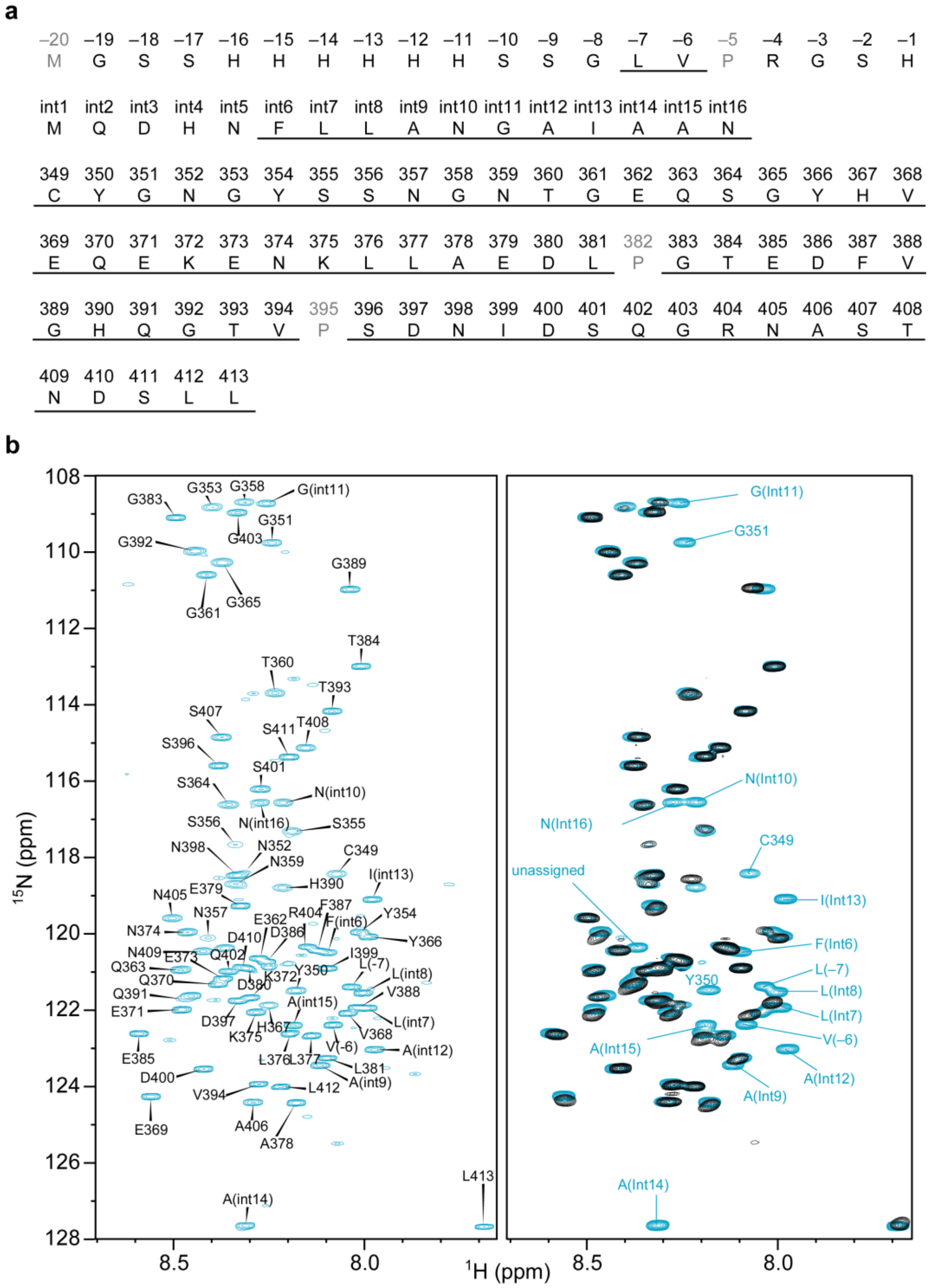

Supplementary Figure 2 Assignment of the ^{1}H-^{15}N HSQC spectrum of IntC-Cterm. (a) Amino acid sequence of Int_C-Cterm. Assigned residues are underlined. (b) ^{1}H-^{15}N HSQC spectrum of [u-^{2}H, ^{13}C, ^{15}N] Int_C-Cterm, with the assignment (left) and overlay of the ^{1}H-^{15}N HSQC spectra of [u-^{2}H, ^{13}C, ^{15}N] Int_C-Cterm (cyan) and {Cterm- [^{2}H, ^{13}C, ^{15}N]} β_2AR in rHDLs (black). The resonances not observed in the spectra of β_2AR in rHDLs are indicated.

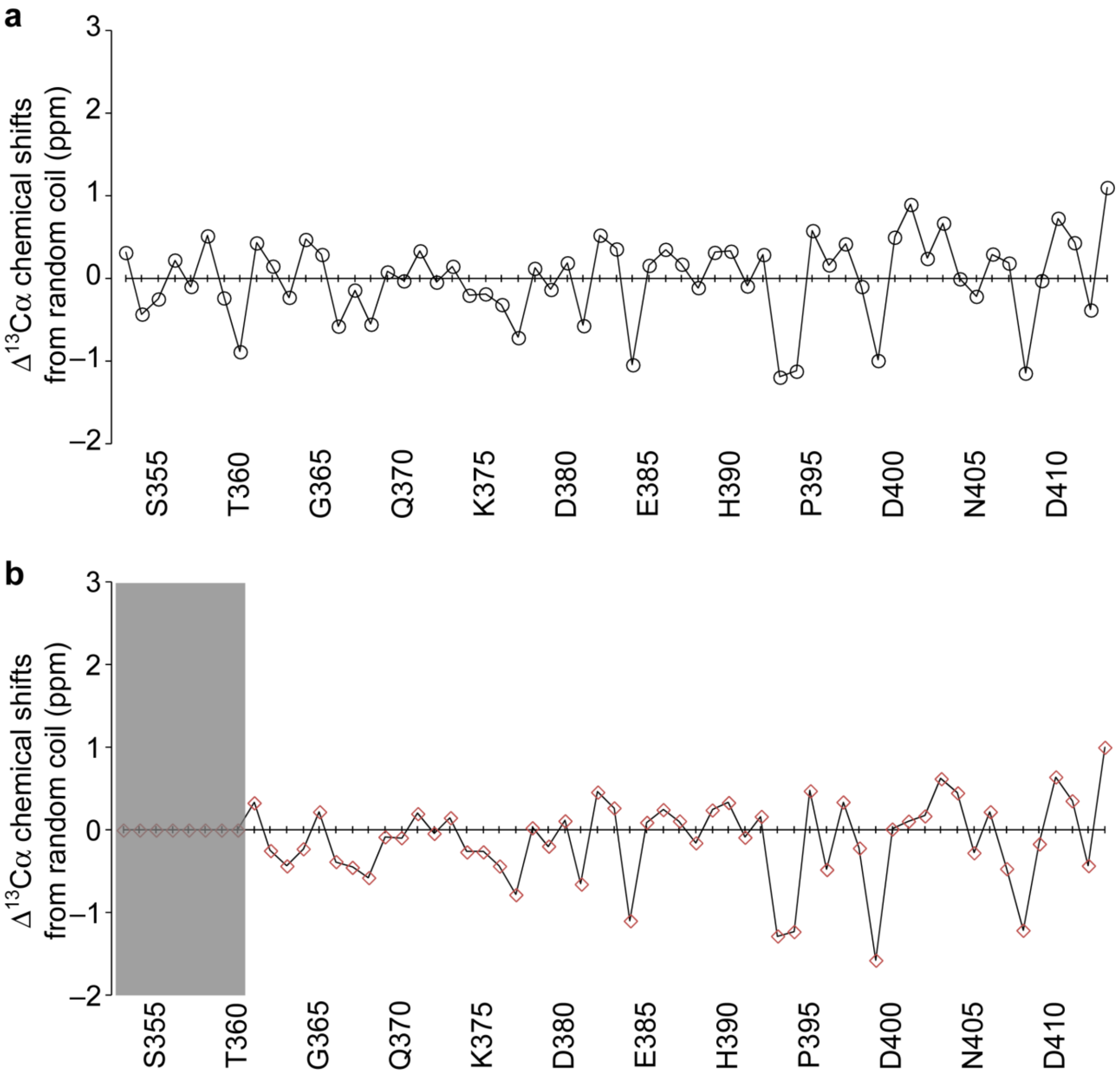

Supplementary Figure 3 Secondary structure of the C-terminal region of β_2AR.

Differences of the Cα chemical shifts observed in the {Cterm- [^{2}H, ^{13}C, ^{15}N]} β_2AR in rHDLs in the unphosphorylated state (a) and phosphorylated state (b) from the Cα chemical shifts in a random coil. Resonances from G353-T360 of the phosphorylated β_2AR were not observed.

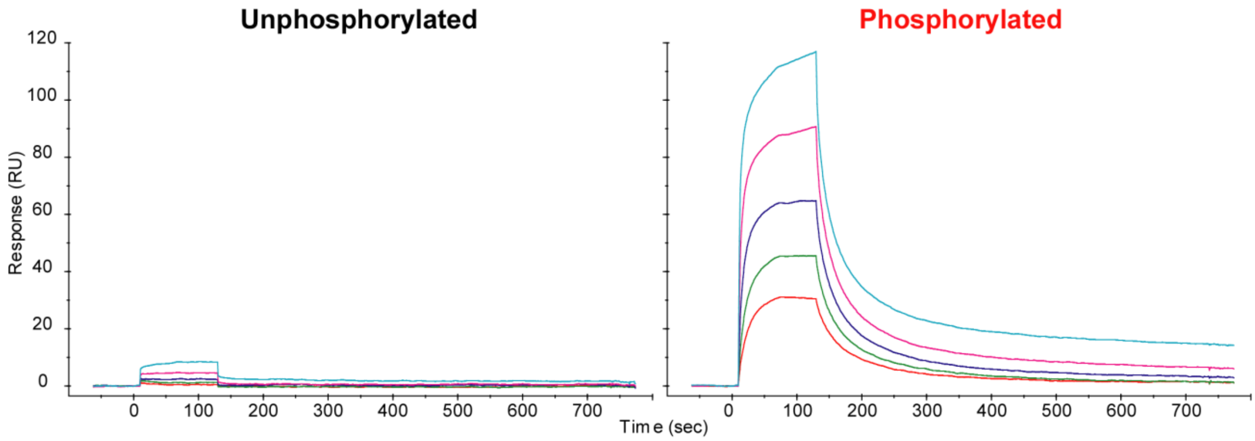

Supplementary Figure 4 SPR analyses of the interaction between β-arrestin 1 and β_2AR embedded in rHDLs. Overlay of the sensorgrams obtained for the interaction between 31 ~ 500 nM β-arrestin 1 and immobilized unphosphorylated (left) or phosphorylated (right) β_2AR mutant embedded in rHDLs, in the full agonist-bound state.

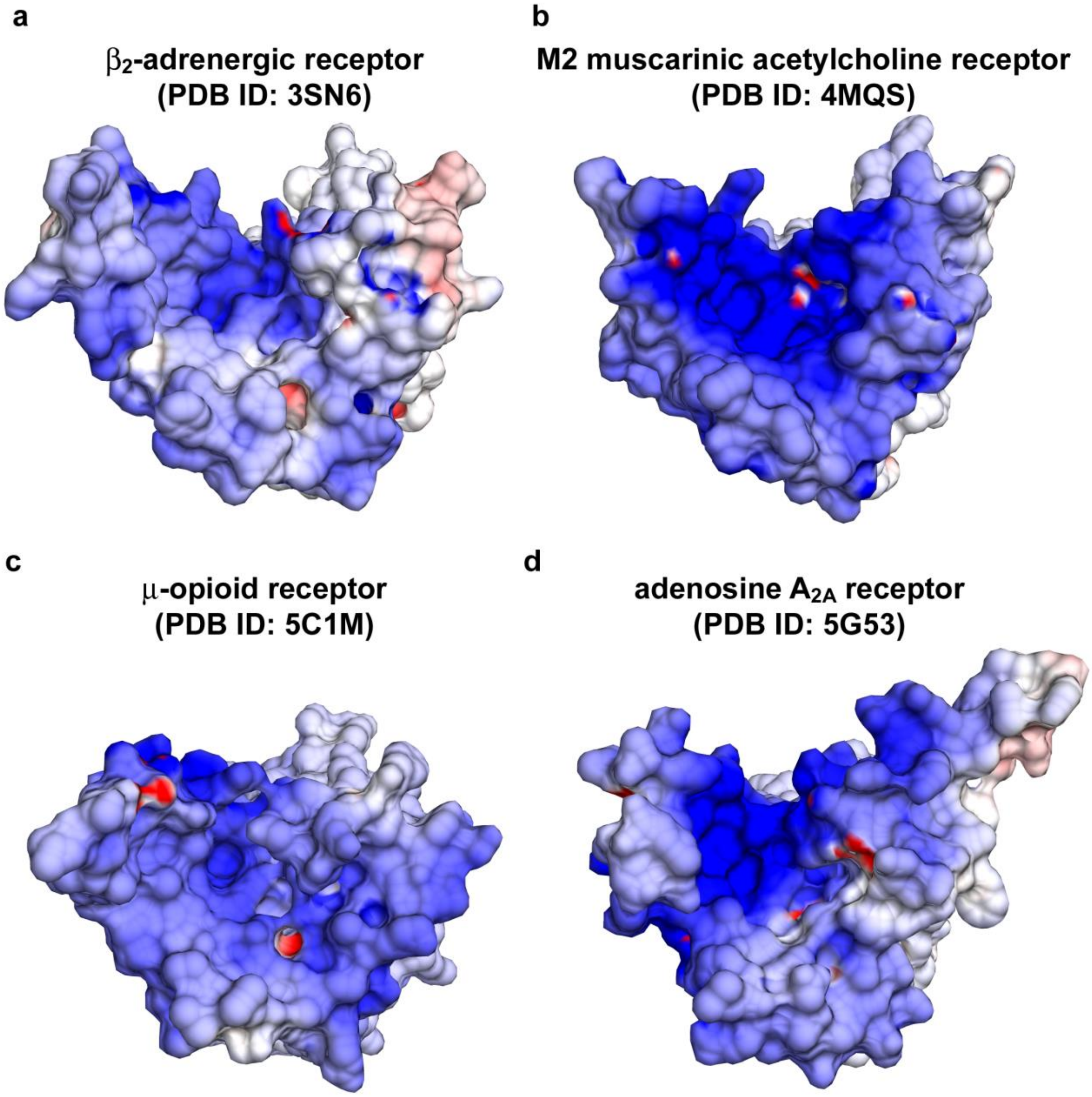

Supplementary Figure 5 Positive charge propensities of the cytoplasmic faces of GPCRs in active conformations. Surface charge potentials of the cytoplasmic faces of GPCRs in the active conformations: β_2AR[2], PDB ID: 3SN6 (a); M2 muscarinic acetylcholine receptor[3], PDB ID: 4MQS (b); μ-opioid receptor[4], PDB ID: 5C1M (c); adenosine A_{2A} receptor[5], PDB ID: 5G53 (d). Addition of hydrogen atoms and assignments of charge and radius parameters were executed using PDB2PQR[6]. Electrostatic potentials were calculated using APBS[7]. Positive and negative charge potentials are shown in blue and red, respectively.

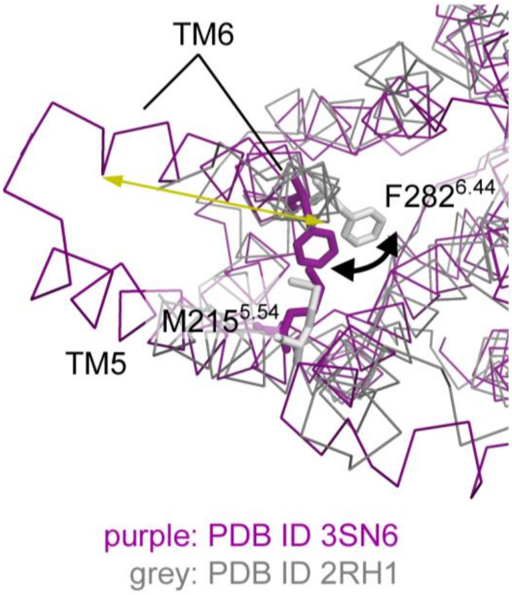

Supplementary Figure 6 Conformational change around M215$^{5.54}$ and TM 6 upon activation. The crystal structures of β_2AR with an inverse agonist, carazolol (PDB ID: 2RH1)[8] (grey) and with a full agonist, BI-167107, and a G-protein (PDB ID: 3SN6)[2] (purple) are overlaid and shown in a cytoplasmic view. TM helices are depicted by Cα traces, and M215$^{5.54}$ and F282$^{6.44}$ are depicted by stick models. In the inverse agonist-bound state, the aromatic ring of the F282$^{6.44}$ side chain is farther away from the methyl group of the M215$^{5.54}$ side chain. In contrast, in the full agonist and G-protein-bound state, the aromatic ring of the F282$^{6.44}$ side chain approaches the methyl group of the M215$^{5.54}$ side chain, and TM6 moves outward. The structural model was prepared with Cuemol (http://www.cuemol.org/).

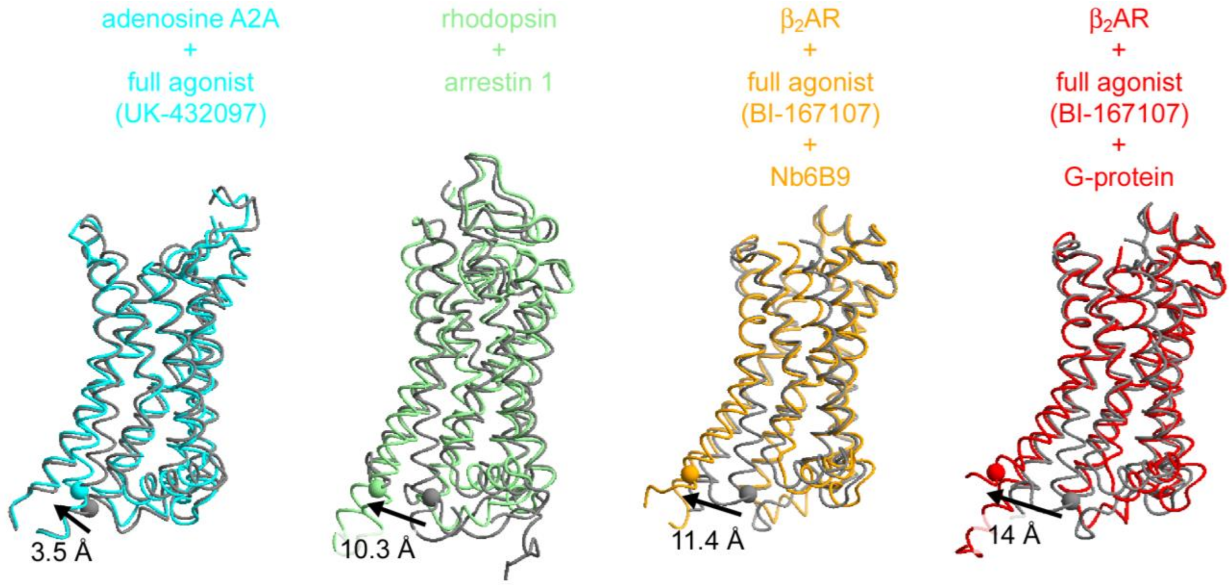

Supplementary Figure 7 The degrees of the outward movement of TM6 in various active states. The outward movements of TM6 were measured at $Glu^{6.30}$ Cα between the inactive- and active-conformation structures. The inactive state structures are depicted by grey tubes[8-10]. The active state structures of adenosine A_{2A}[11], rhodopsin bound to arrestin 1[12], β_2AR bound to Nb6B9[13], and β_2AR bound to G-protein[2] are depicted by cyan, green, orange, and red tubes, respectively. The Cα carbons of $Glu^{6.30}$ are depicted by spheres. The PDB IDs of the structures are as follows: inactive adenosine A_{2A}: 3EML, active adenosine A_{2A}: 3QAK, inactive rhodopsin: 1F88, active rhodopsin bound to arrestin 1: 4ZWJ, inactive β_2AR: 2RH1, active β_2AR bound to Nb6B9: 4LDE, active β_2AR bound to G-protein: 3SN6.

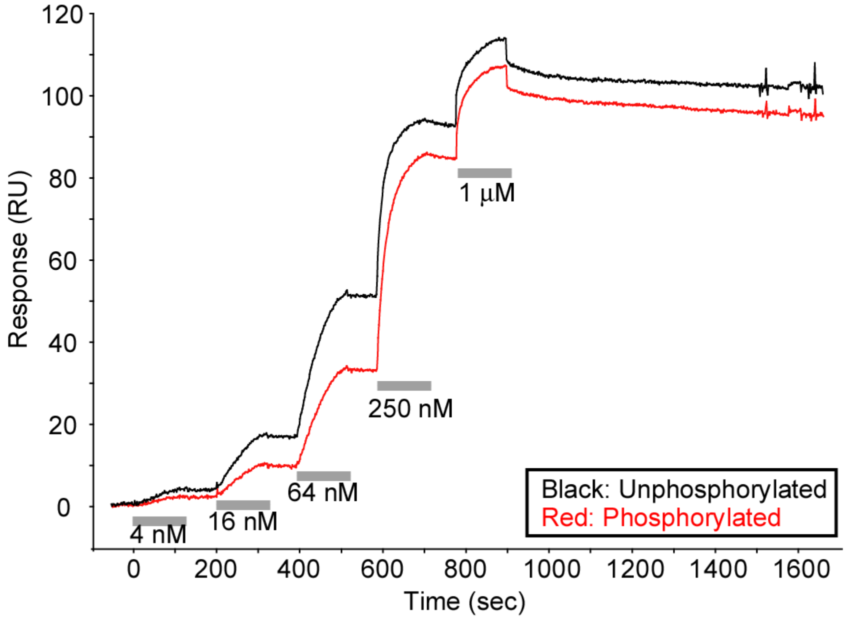

Supplementary Figure 8 SPR analyses of the interaction between Nb6B9 and β2AR embedded in rHDLs. Sensorgrams obtained for the interaction between 4 ~ 1,000 nM Nb6B9 and immobilized unphosphorylated (black) or phosphorylated (Red) β2AR embedded in rHDLs, in the full agonist-bound state.

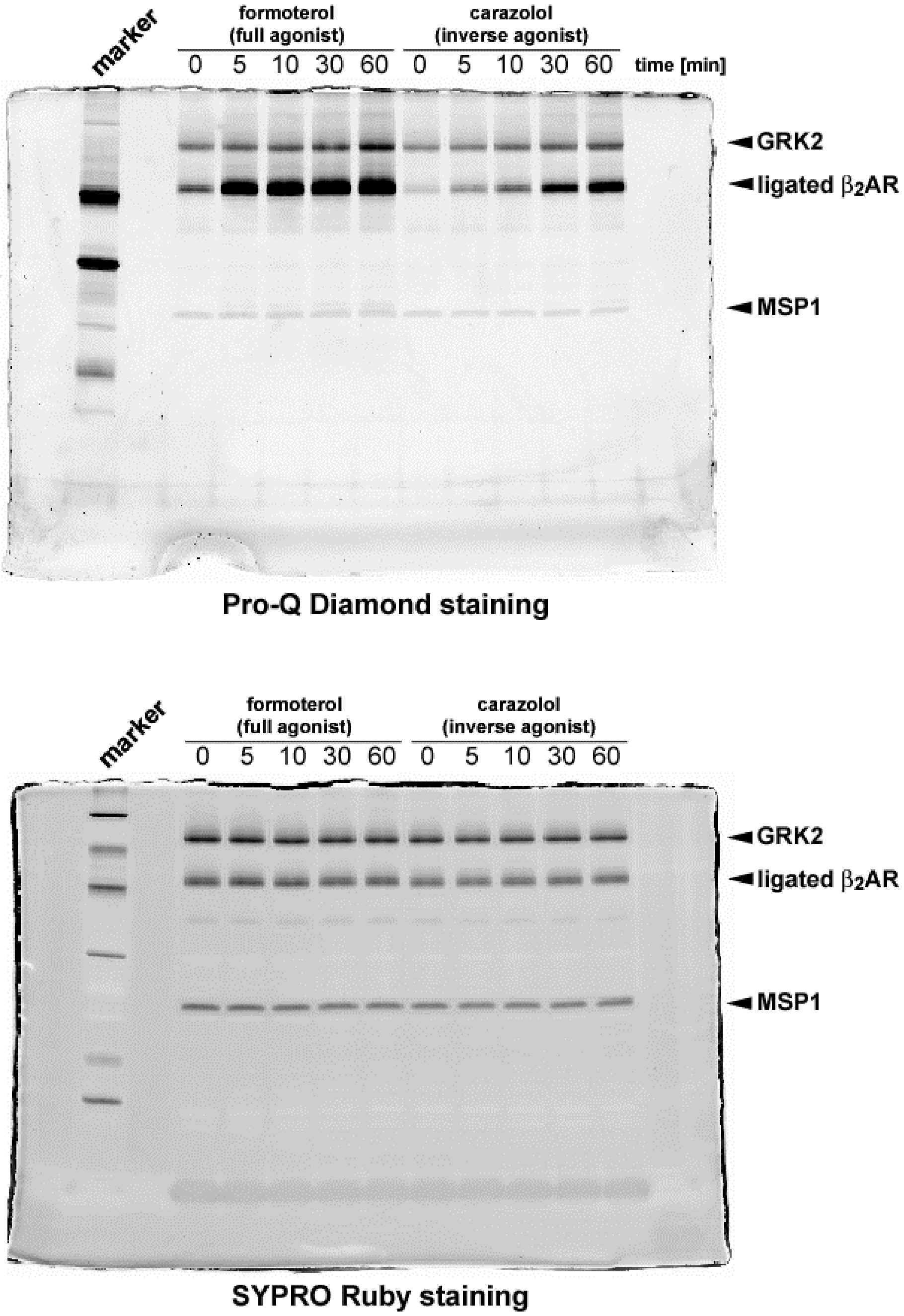

Supplementary Figure 9 **Uncropped images of the gels in Supplementary Figure 1c**

Supplementary Table 1 The degrees of GRK2-mediated phosphorylation on each serine and threonine residue in the C-terminal region

Residue	Full agonist (formoterol)*	Inverse agonist (carazolol)*
**S355/S356**	N.D.	N.D.
**T360**	> 95%	35.6 ± 1.7%
**S364**	> 94.5%	45.4 ± 2.5%
**S396**	35.4 ± 1.0%	9.9 ± 0.2%
**S401**	49.1 ± 1.6%	5.7 ± 0.1%
**S407**	60.2 ± 2.2%	8.4 ± 0.2%

*The degrees of the phosphorylation of each serine and threonine residue, are calculated from the intensity reductions of NMR signals corresponding to the unphosphorylated state upon phosphorylation. Errors are calculated from the root sum square of the (noise level/signal intensity) in the two spectra, before and after phosphorylation.

Supplementary Table 2 Primer sequences used in this study

**β_2AR cloning**	CATGCCATGGGGCAACCCGGGAACGG
	GCTCTAGATTAGTGATGGTGATGGTGATGGTGATGGTGATGCA<br>GCAGTGAGTCATTTGTACTAC
**B2AR E122W mutation**	CACGGCCAGCATTTGGACCCTGTGCGTG
	CACGCACAGGGTCCAAATGCTGGCCGTG
**β_2AR N187E mutation**	GCCATCAACTGCTATGCCGAAGAGACCTGCTGTGACTTC
	GAAGTCACAGCAGGTCTCTTCGGCATAGCAGTTGATGGC
**β_2AR C265A mutation**	CCGCAGATCTTCCAAGTTCGCCTTGAAGGAGCACAAAGCCC
	GGGCTTTGTGCTCCTTCAAGGCGAACTTGGAAGATCTGCGG
**β_2AR M1A mutation**	GGACGATGACGCCGCGGGGCAACCCGGG
	CCCGGGTTGCCCCGCGGCGTCATCGTCC
**β_2AR M96T/M98T mutation**	GCCCATATTCTTACGAAAACGTGGACTTTTGGC
	GCCAAAAGTCCACGTTTTCGTAAGAATATGGGC
**β_2AR M156L mutation**	CGGGTGATCATTCTGCTGGTGTGGATTGTGTC
	GACACAATCCACACCAGCAGAATGATCACCCG
**β_2AR M171S mutation**	CCTTCTTGCCCATTCAGAGCCACTGGTACCGGGCC
	GGCCCGGTACCAGTGGCTCTGAATGGGCAAGAAGG

**Insertion of DnaE intein from *Nostoc punctiform* to β2AR plasmid**	GGTCTTCTTTGAAGTGTTTAAGCTATGAAACGGAAATATTG
	GCCATTCCCATAGGCATTAGAAGCTATGAAGCCATTTTTGAG
**Insertion of Int_C-Cterm**	CATGCCATGGGCAGCAGCCATCATCA
	CCCAAGCTTACAGCAGTGAGTCATTTGTACTAGC
**Insertion of V2R sequence to β2AR plasmid**	CTGCGCAGGTCTTCTTTGAAGGCC
	GCACAGAAGCTCCTGGAAGGCAATCC
	CAGGAGCTTCTGTGCGCCCGGGGACGCACCCCACCCAGCCTGGGTCCCCAAGATGAGTCCTGCACCACCGCCAGC
	AGAAGACCTGCGCAGTCACGATGAAGTGTCCTTGGCCAGGGAGGAGCTGGCGGTGGTGCAGGACTCATCTTGGGG
**β2AR C378A mutation**	GAAAGAAAATAAACTGCTGGCTGAAGACCTCCCAGGCAC
	GCGCCTGGGAGGTCTTCAGCCAGCAGTTTATTTTCTTTC
**β2AR C406A mutation**	GATTCACAAGGGAGGAATGCTAGTACAAATGACTCACTGC
	GCAGTGAGTCATTTGTACTAGCATTCCTCCCTTGTGAATC
**β2AR T384A mutation**	GACCTCCCAGGCGCGGAAGACTTTGTG
	CACAAAGTCTTCCGCGCCTGGGAGGTC
**β2AR T393A/S396A mutation**	GGGCCATCAAGGTGCTGTGCCTGCCGATAACATTGATTC

	GAATCAATGTTATCGGCAGGCACAGCACCTTGATGGCCC
**β_2AR S401A mutation**	GCCGATAACATTGATGCACAAGGGAGGAATGC
	GCATTCCTCCCTTGTGCATCAATGTTATCGGC
**β_2AR S407A/T408A/S411A mutation**	CAAGGGAGGAATGCTGCTGCAAATGACGCACTGCTGTAAGC
	GCTTACAGCAGTGCGTCATTTGCAGCAGCATTCCTCCCTTG
**β-arrestin 1 cloning**	CTAGCTAGCATGGGCGACAAAGGGACCCGAG
	GCCAAGCTTATCTGTTGTTGAGCTGTGGAG
**β-arrestin 1 TEV protease site insertion**	CATGGTATGGCTAGCGAGAATCTCTATTTTCAGGGCATGGGCGACAAAGGG
	CCCTTTGTCGCCCATGCCCTGAAAATAGAGATTCTCGCTAGCCATACCATG
**β-arrestin 1 C59V mutation**	CTATGTGACGCTGACCGTCGCCTTCCGCTATGGC
	GCCATAGCGGAAGGCGACGGTCAGCGTCACATAG
**β-arrestin 1 C125S mutation**	CCCTCCAAACCTTCCATCTTCTGTGACACTGCAG
	CTGCAGTGTCACAGAAGATGGAAGGTTTGGAGGG
**β-arrestin 1 C140L/C150L mutation**	CGGGGAAGGCTTTGGGTGTGGACTATGAAGTCAAAGCCTTCGTCGCGGAGAATTTGG
	CCAAATTCTCCGCGACGAAGGCTTTGACTTCATAGTCCACACCCAAAGCCTTCCCCG

**β-arrestin 1 C242V/C251V mutation**	GTATGCAGACATCGTCCTTTTCAACACAGCTCAGTACAAGGTCCCTGTTGCC
	GGCAACAGGGACCTTGTACTGAGCTGTGTTGAAAAGGACGATGTCTGCATAC
**β-arrestin 1 C269S mutation**	CCCAGCTCGACGTTCAGCAAGGTCTACACACTG
	CAGTGTGTAGACCTTGCTGAACGTCGAGCTGGG

Supplementary Note 1

Phosphorylation of the C-terminal region examined by NMR

The ^{1}H-^{15}N HSQC spectrum of β_2AR phosphorylated by GRK2 in the presence of the full agonist revealed the remarkable ^{1}H downfield shifts at the resonances from S364, S396, S401, and S407 as well as the resonances from residues with chemical shifts almost identical to those of unphosphorylated β_2AR (Fig. 1). The former and latter resonances are derived from the phosphorylated and unphosphorylated residues, respectively, because the amide protons of phosphorylated serine and threonine residues exhibit ^{1}H downfield shifts, due to the hydrogen bonds between the phosphate and the amide groups[1]. According to the signal intensities of the resonances from the phosphorylated and unphosphorylated residues, 35~95% of these residues are phosphorylated by GRK2 (Supplementary Table 1).

Resonances from S355, S356, and T360 were not observed, suggesting that these signals were broadened due to the exchange between the multiple conformations in the phosphorylated state. In the ^{1}H-^{13}C HMQC spectrum of the phosphorylated β_2AR, a resonance from T360 was observed with the ^{13}C chemical shift typical at a phosphorylated threonine residue [1] (Fig. 1). Therefore, > 90% of T360 was phosphorylated by GRK2 (Supplementary Table 1).

The populations of phosphorylated residues close to the TM region were higher than those distal to the TM region. In β_2AR phosphorylated in the presence of the inverse agonist, the population of phosphorylated residues was remarkably lower than that in β_2AR

phosphorylated in the presence of the full agonist (Supplementary Table 1). This is in agreement with the SDS-PAGE experiments (Supplementary Fig. 1).

Supplementary References

1. Bienkiewicz, E.A. & Lumb, K.J. Random-coil chemical shifts of phosphorylated amino acids. *J. Biomol. NMR* **15**, 203-6 (1999).
2. Rasmussen, S.G. et al. Crystal structure of the β_2 adrenergic receptor-Gs protein complex. *Nature* **477**, 549-55 (2011).
3. Kruse, A.C. et al. Activation and allosteric modulation of a muscarinic acetylcholine receptor. *Nature* **504**, 101-6 (2013).
4. Huang, W. et al. Structural insights into μ-opioid receptor activation. *Nature* **524**, 315-21 (2015).
5. Carpenter, B., Nehmé, R., Warne, T., Leslie, A.G. & Tate, C.G. Structure of the adenosine A_{2A} receptor bound to an engineered G protein. *Nature* **536**, 104-7 (2016).
6. Dolinsky, T.J., Nielsen, J.E., McCammon, J.A. & Baker, N.A. PDB2PQR: an automated pipeline for the setup of Poisson-Boltzmann electrostatics calculations. *Nucleic Acids Res.* **32**, W665-7 (2004).
7. Baker, N.A., Sept, D., Joseph, S., Holst, M.J. & McCammon, J.A. Electrostatics of nanosystems: application to microtubules and the ribosome. *Proc. Natl. Acad. Sci. USA* **98**, 10037-41 (2001).
8. Rosenbaum, D.M. et al. GPCR engineering yields high-resolution structural insights into β_2-adrenergic receptor function. *Science* **318**, 1266-73 (2007).
9. Palczewski, K. et al. Crystal structure of rhodopsin: A G protein-coupled receptor.

Science **289**, 739-745 (2000).

10. Jaakola, V. et al. The 2.6 angstrom crystal structure of a human A_{2A} adenosine receptor bound to an antagonist. *Science* **322**, 1211-1217 (2008).

11. Xu, F. et al. Structure of an agonist-bound human A_{2A} adenosine receptor. *Science* **332**, 322-327 (2011).

12. Kang, Y. et al. Crystal structure of rhodopsin bound to arrestin by femtosecond X-ray laser. *Nature* **523**, 561-7 (2015).

13. Ring, A. et al. Adrenaline-activated structure of β_2-adrenoceptor stabilized by an engineered nanobody. *Nature* **502**, 575-+ (2013).
